# Microstructural tissue-engineering in the rachis and barbs of bird feathers

**DOI:** 10.1038/srep45162

**Published:** 2017-03-27

**Authors:** Theagarten Lingham-Soliar

**Affiliations:** 1 Nelson Mandela Metropolitan University, Coastal and Marine Research, South Campus, University Way, Port Elizabeth, 6001, South Africa; 2 University of KwaZulu-Natal, Life Sciences, Westville Campus, Durban 4000, South Africa

## Abstract

Feathers do not have to be especially strong but they do need to be stiff and at the same time resilient and to have a high work of fracture. Syncitial barbule fibres are the highest size-class of continuous filaments in the cortex of the rachis of the feather. However, the rachis can be treated as a generalized cone of rapidly diminishing volume. This means that hundreds of syncitial barbule fibres of the rachis may have to be terminated before reaching the tip – creating potentially thousands of inherently fatal crack-like defects. Here I report a new microstructural architecture of the feather cortex in which most syncitial barbule fibres deviate to the right and left edges of the feather rachis from far within its borders and extend into the barbs, side branches of the rachis, as continuous filaments. This novel morphology adds significantly to knowledge of β-keratin self-assembly in the feather and helps solve the potential problem of fatal crack-like defects in the rachidial cortex. Furthermore, this new complexity, consistent with biology’s robust multi-functionality, solves two biomechanical problems at a stroke. Feather barbs deeply ‘rooted’ within the rachis are also able to better withstand the aerodynamic forces to which they are subjected.

Feathers of flying birds are subjected to extraordinary aerodynamic forces during flight^1^. They are made of a remarkably hard material, keratin. During the first half of the last century pioneering X-ray studies^2^,^3^ indicated that the conformation of the polypeptide chain in the hard keratins of birds and reptiles is based on the β-pleated-sheet (β-form) rather than the coiled-coil α-helix (α-form) found in mammalian keratins. Early use of transmission electron microscopy (TEM) on chicken and seagull feather rachises showed that β-keratin was composed of a framework of fine microfibrils approximately 30 angstroms (Å) in diameter^4^ and that these long protein filaments were surrounded by an amorphous protein matrix, each filament possessing a helical structure with four repeating units per turn^5^,^6^,^7^. Ultrastructural studies next turned to the developmental biology of the feather including, perhaps the most controversial question, the origin and evolution of the feather^8^,^9^,^10^,^11^,^12^,^13^,^14^,^15^,^16^.

β-keratin is the toughest natural polymer known^17^ (toughness can be defined as the quantity of energy required to break or fracture a given cross-section of material–see below). So as to understand the nature of this toughness of feathers, my own work focused on the microstructure (micron level of investigation) of the feather rachis and barbs^18^,^19^. The cortex makes up the upper and lower structural surfaces of the rachis and barbs and has been shown to account for most of its tensile stiffness (modulus of elasticity, see below)^20^. That stiffness is achieved by the efficiency of the bond between the amorphous polymer matrix and the polymeric filaments of β-keratin. It is precisely the efficiency of this bond that has circumvented details of feather microstructure by conventional structure determination methods. Histodifferentiation and TEM^13^ have produced important answers in feather molecular structure and developmental biology but my attempt to resolve questions at the microstructural level of fibre hierarchy needed a new form of investigation. This involved lab-based microbial hydrolysis of the amorphous polymer matrix of the rachidial cortex^18^,^19^,^21^ which in turn freed or delineated for the first time the highest fibre size-class of the feather rachis, the syncitial barbule fibres (SBFs hereafter). The SBFs are comprised of sub-fibres (hereafter microfibrils^4^ or fibrils^18^,^19^). This new SBF hierarchy was graphically shown by scanning electron microscopy (SEM)^18^. The SBFs are several magnitudes greater in size than the microfibrils previously identified in the cortex of the feather rachis^4^. SBFs form long, continuous filaments of β-keratin, the majority of which are tightly assembled parallel to the longitudinal axis of the rachis, in 2- and 3-D planes. Subsequently, the same SBF hierarchy was revealed in the cortex of the dorsal and ventral walls of the barbs^19^.

Perhaps one of the most intriguing questions in bird flight involves how toughness or a high work of fracture is achieved in the cortex of the rachis and barbs^19^,^22^. Put another way, what are the material conditions that would help prevent (or delay) the feather from splitting or cracking down its length or across its hoop (circumference) during the stresses of flight. That question was intuitively first raised over 38 years ago by the notable aeronautical engineer, John Gordon^23^ although he declared it was a mystery at the time of writing. However, recent research on the cortical SBF structure of the rachis and barbs has allowed new light to be shed on the problem^18^,^19^,^22^. But, as so often happens as we find new answers we also find more questions. I consider one such question here, which is also closely tied to bird flight.

Historically most of the fibres of the feather rachis (apart from a thin band or two aligned around the hoop) were considered to be aligned along the longitudinal axis^4^,^19^,^24^,^25^,^26^. This contrasts with proposals in a recent study^27^ in which the authors suggest that the rachidial cortex is comprised of multiple laminae of differentially oriented fibres. This will be mentioned briefly in my discussion below.

The rachis has a general morphology of a tapering cone^28^,^29^ (Fig. 1a), while in actuality its precise cross-sectional shape is four-sided, square to rectangular^15^,^19^,^28^. This generalized attenuated cone entails linearly decreasing volumes of the rachidial cortex from base to tip as shown definitively by X-ray diffraction analysis^29^ – volume greater proximally compared to distally (Fig. 1b). The question that arises, how is a system of continuous β-keratin SBFs extending in the proximo-distal direction of the rachidial cortex^18^,^19^ accommodated by a linearly decreasing volume of the cortex, given that there are thousands of SBFs that comprise the dorsal and ventral cortices of the feather rachis^18^?

One way would be for hundreds of SBF terminations in the proximo-distal direction of the rachis. However, I propose that if excess SBFs are simply terminated in the cortex along the rachis length it would result in notches or free ends that would locally concentrate the stress at each fibre tip. Simple mechanics shows that sudden failure in a material begins at a notch or crack that locally concentrates the stress^23^. This is analogous to the scissor-snip a tailor makes before tearing a piece of fabric. In the 1920 s, Griffith^30^ showed that according to thermodynamic principles the magnitude of the stress concentration at a crack tip is dependent on the crack length (*L*) i.e. that the strain energy released in the area around the crack length (*L*^2^, proportional to the crack length) is available for propagating the crack. From this principle we see that there is a dangerous potential of numerous self-perpetuating cracks in the feather cortex. How then have birds in the 150 million years of their evolution been able to respond to this threat ? The present hypothesis is that there must be a means to eliminate this potentially catastrophic condition in a structure critical to bird flight – i.e., crucially a structural mechanism to avoid an inherent condition of notches or cracks in the feather rachidial cortices. To this end the feather cortices and comprising SBFs are investigated.

## Results and Descriptions

### Syncitial barbule fibres

**Figure 1:**
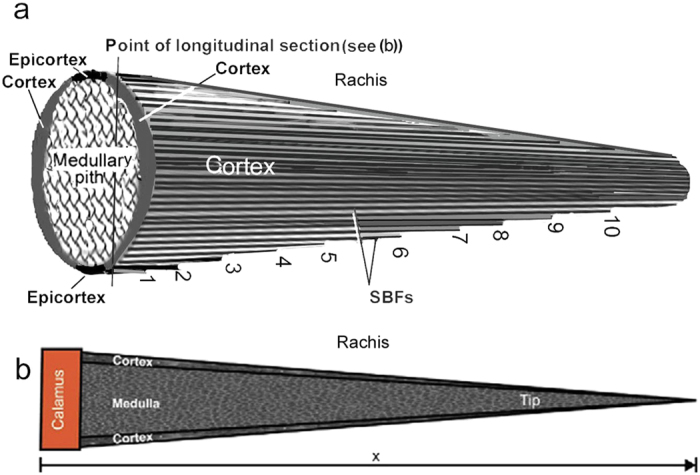
Feather rachis as a cone. (**a**) Diagrammatic view of the rachis as a tapering cone showing potential terminations of SBFs (numbered 1–10) because of the linear decrease in cortex thickness in the proximo-distal direction. (**b**) Diagram of rachis of *Pavo cristatus* sub. alba, subjected to X-ray diffraction along its length, showing that both outer diameter and cortex thickness decrease linearly from the base to the tip, modified^29^.

**Figure 2:**
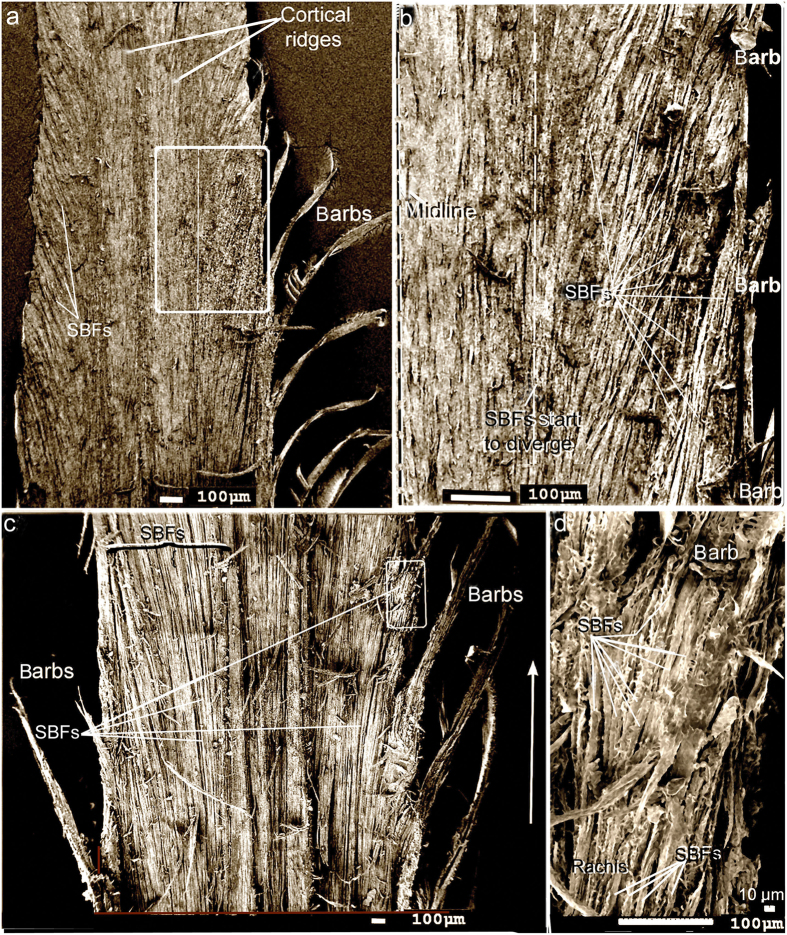
SEM of primary flight feathers Peeled longitudinal section (see Material and methods) from rachis cortex mid-length area. (**a**) and (**b**), sacred ibis, *Threskiornis aethiopicus*. (**a**) Section of rachis directly level with the barbs. Approximately one third of the SBFs on each side of the central region diverge toward the barbs. (**b**) Detail of area demarcated in (**a**). Approximately ⅓ SBFs on either side of the rachis diverge. The black eagle, *Aquila verreauxii*. (**c**) SEM of SBFs diverging en masse (~⅔) to the barbs on both sides of the rachis. (**d**) Detail at barb rachis interface of SBFs entering the barbs from the rachis. White arrow represents the longitudinal axis in all sub-figures.

**Figure 3:**
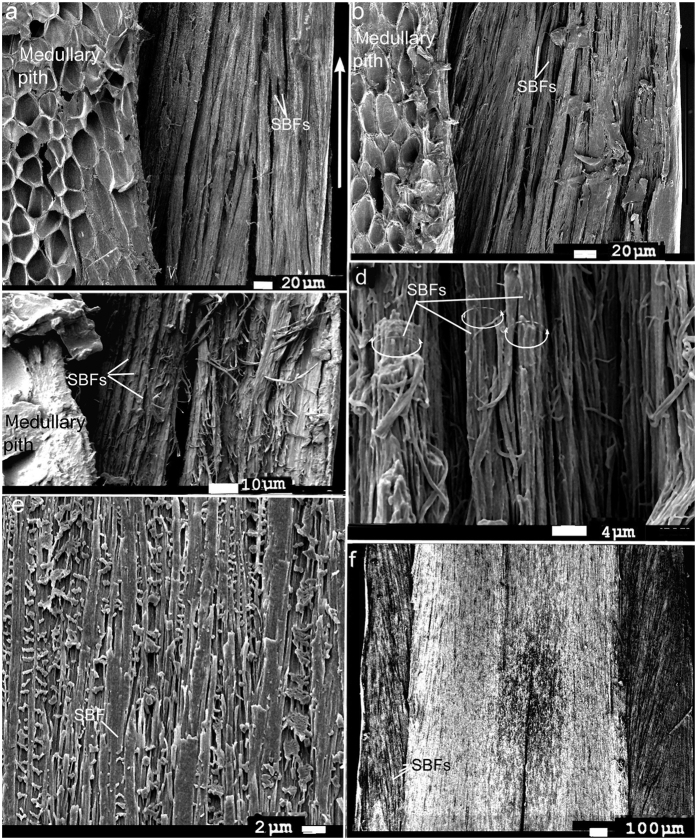
Sem. Primary flight feather. Rachis (~mid-length). All longitudinally peeled sections except (**e**). (**a–c**) SBFs deep in the cortex, adjacent to medullary pith. SBFs diverge from the right quarter of the rachis toward its edge where they reorient to the rachis long axis. (**a**) Scarlet ibis, *Eudocimus ruber.* (**b**) Marsh harrier, *Circus ranivorus*, (**c**) Pygmy falcon, *Polihierax semitorquatus*. (**d**) Blue and yellow macaw, *Ara ararauna,* left side of rachis (SBFs surface treated with solution of acetone to reveal fibrils). Hemicircles show approximate thickness of the SBFs. (**e**) Mute swan, *Cygnus olor*. (microtomed section).Traces of the syncitial barbules are evident although most are cut through. (**f**) Spur-winged goose, *Plectropterus gambensis*. SBFs near the surface of the cortex on either side of the rachis, comprising about half the rachis width in total. Arrow represents the longitudinal axis in all sub- figures.

**Figure 4:**
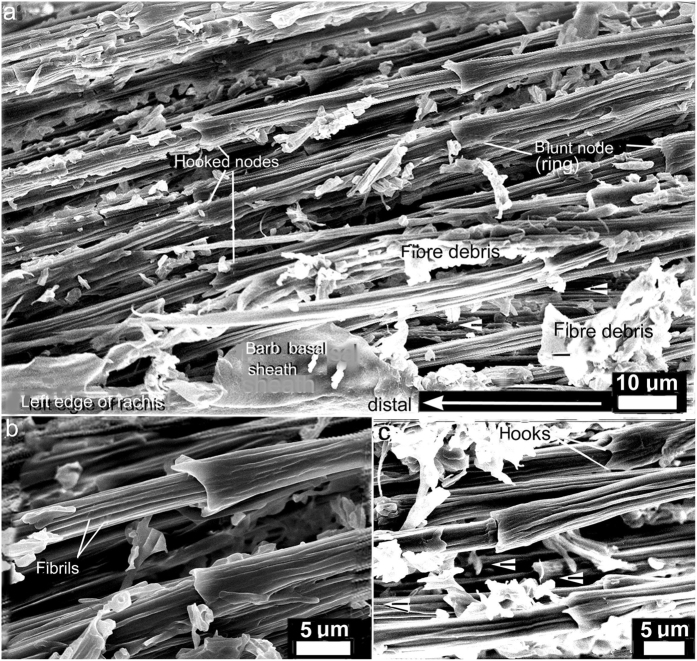
SEM. Primary flight feather. Rachis cortex. Native feather of *Falco tinnunculus*, sectioned manually using a blade. (**a**) SBFs from the cortex in the mid-length of the rachis, on the left side just adjacent to and level with the barbs (evidenced by the barb basal sheaths/petioles). There are ~6 layers of SBFs all similarly oriented except along the left edge where the SBFs run straight (arrowheads). Numerous SBF nodes both with and without hooks can be seen. Most SBFs show inner fibrils the next level down in the fibre hierarchy. Note, some SBFs were dissected (as noted by SBF debris) or pulled out. Top left, shows ~3 SBF layers still tightly ‘glued’ together. (**b**) Fine detail from an area in (**a**). (**c**) A section just to the right of the area in (**a**), showing both hooked and ringed nodes of SBFs. Arrow represents the longitudinal axis in all images.

**Figure 5:**
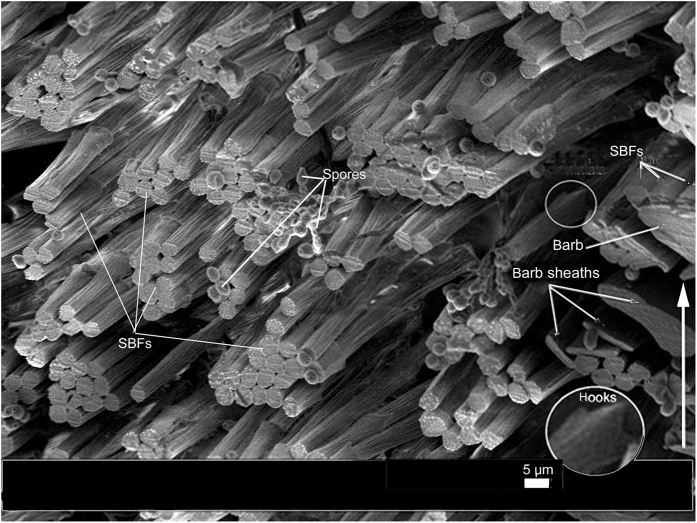
SEM. *Gallus gallus*, primary flight feather, mid-rachis, cortex fungal delineated showing hundreds of SBFs. Oblique (part transverse- longitudinal)-section representing a large part of the upper cortex, ± one third total depth and ± one quarter of cortex width (on the right side). On far right can be seen dorsal part of transected barb with SBFs exposed at cortical surface. Below barb are trans-sectioned barb sheaths/petioles. Inset (circle) shows SBF node with hooks. White arrow represents the longitudinal axis.

**Figure 6:**
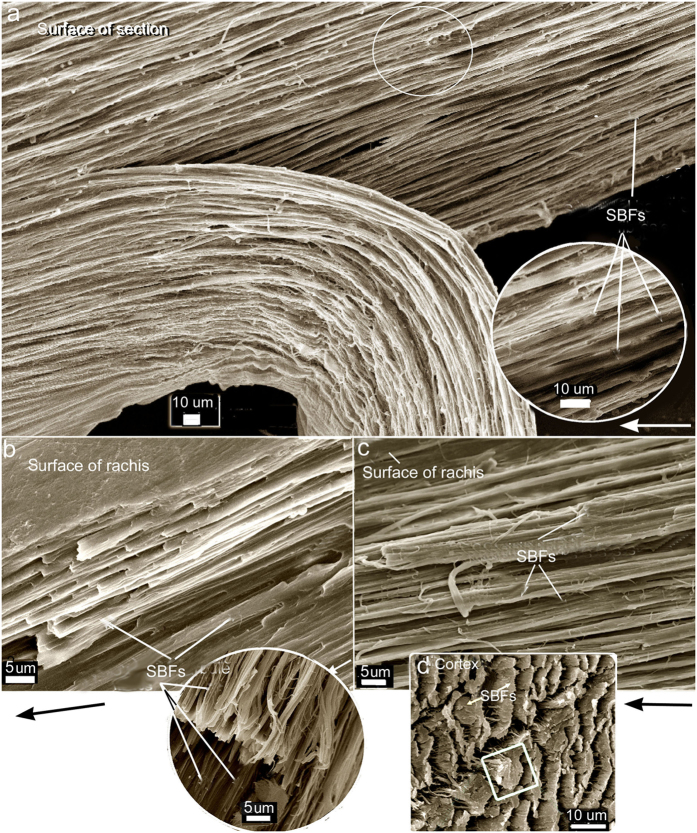
SEM of longitudinal and transverse sections of SBFs of the rachidial cortex. (**a–c**), flight feathers, left side of rachis. (**a**) Block of deep longitudinal section of partially fungal degraded (early) rachidial cortex of *Gallus gallus* with a thick part peeled back to reveal numerous layers (~20) of SBFs all oriented parallel to each other at a constant angle (section taken at ¾ rachis length and representing ± 2/3 cortical depth). Circled area enlarged in the inset. (**b**) Freeze-fractured partial vertical section of the rachidial cortex of native feather (mid-length) of *Falco tinnunculus* exposing from the surface ~10 layers of identically oriented SBFs along the longidutinal axis. Inset circle shows an oblique cross-section (cross-longitudinal) showing SBFs oriented identically (~8 layers). (**c**) Freeze-fractured section similar to (**b**) of *Falco peregrinus* showing ~10 layers of identically oriented SBFs along the longidutinal axis. Arrows in (**a**), (**b**) and (**c**) show longitudinal axis of rachis. (**d**) Tail feather of the rachidial cortex (ventral, distal) of the Toco toucan (*Ramphastos toco*) (freeze-fractured transverse section), modified^26^, showing the SBFs similarly oriented in several layers (square, outlines a single SBF).

**Figure 7:**
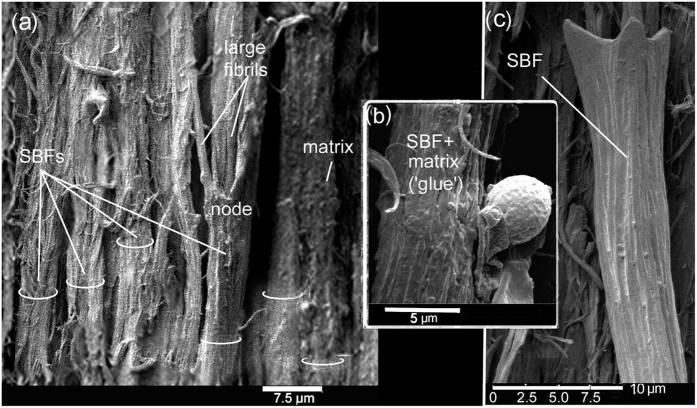
SEM. Primary flight feather of *Polihierax semitorquatus*. Cortex. Section longitudinally peeled at ~mid-rachis length. Right side. (**a**) The partial stripping of the SBFs by the peeling process shows *in situ* the dynamic nature of SBF cohesion and compaction and how bonding is achieved by just a thin layer of matrix. On the right, two SBFs have bonded almost into one. Hemi-circles define thickness of some SBFs. (**b**) Presence of a small infestation of fungi. The matrix is still intact and un-degraded showing that there is little additional thickness compared to a degraded SBF (**c**). (**c**) Close by (to the left of (**a**) fungi have completely stripped a SBF of its matrix, showing the typical structure.

**Figure 8:**
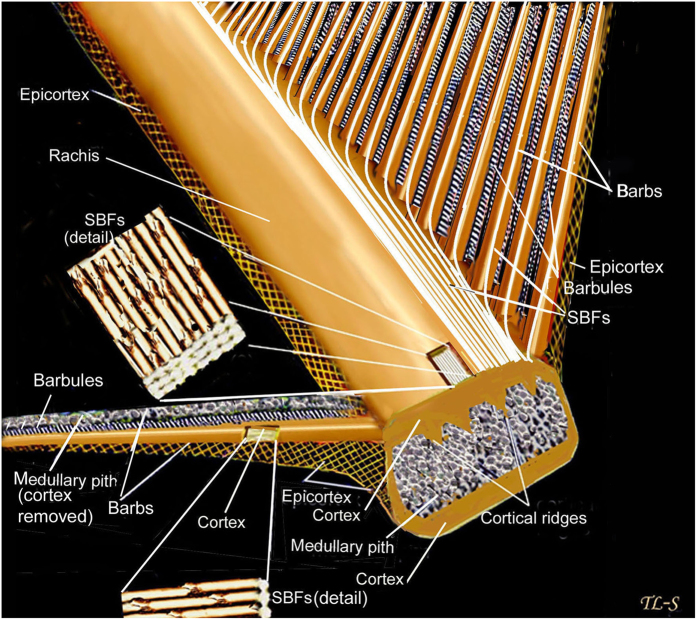
Schematic view of the rachis and barbs. A diagrammatic representation of an uninterrupted architecture between the rachis and barbs comprising masses of cortical SBFs. For simplicity it is best visualized as single diverging SBFs (rachis to barbs) from a single layer of the cortex (see Fig. 1). The right side of the rachis only is figured in this context. Figure modified^19^.

Essential to our understanding of the present findings is a keen understanding of the β-keratin SBFs (see Figs 2, 3, 4, 5, 6, 7, 8 and Supplementary Information (SI) Figs 1–5 for both wide-angled and detailed views). SBFs were first discovered in the feather rachis by Lingham-Soliar *et al*.^18^ and subsequently identified by Bode *et al*.^26^ Structurally, the importance of SBFs is implicit given that they form the bulk of the physical and chemical makeup of the rachis and barbs as concentrated in their cortices. The cortex forms both the dorsal and ventral walls of the feather rachis and barbs^18^,^19^ (Figs 5 and 6). Each SBF is a complex fibre and presents a number of key morphological characters: (i) thickness of a single SBF ranges with a diameter of about 5–8 microns along its length which includes, (ii) a relatively slender stem, made up of fibrils (next in order ~0.5–1.0 micron diameter), with (iii) regularly-spaced thickened nodes, furnished with (iv) hooks and/or rings, which in entirety (v) form a continuous filamentous structure that is, (vi) tightly assembled with thousands of others within a matrix to make up the cortices of the rachis and barbs (Figs 2 and 4, 5, 6, 7, 8 and SI Figs 1–3, 5).

The present results show a new structural organization of the SBFs of the rachidial cortex. Rather than the majority of these SBFs being organized strictly parallel to the long axis of the rachis, I report a new, distinctive architecture of the SBFs. SEMs show that the majority of the SBFs of the cortex deviate or divert, in close regularity, to the right and left margins of the rachis and enter the barbs. This was demonstrated in several regions along the rachis and at different depths (Figs 2, 3, 4, 5, 6 and and SI Figs 1, 2g, 5). SBFs that deviate to the left and right margins of the rachis involve ~50–65% of the total SBFs in the entire cortical width. Only in the central area of the cortex and in a thin region along the edges/surfaces of the rachis are the SBFs oriented with the rachidial longitudinal axis (Figs 2 and 3a,b; microstructural details in Figs 4, 5, 6 and SI Fig. 3). SBF angles range from 75.6 to 82.1° increasing proximo-distally (Table 1, SI Fig. 1a–c), which coincide with the increasing barb angles^31^,^32^. These angles are maintained, in numerous sections observed, in the layers at each of these points along the longitudinal axis (Figs 4a, 5 and 6a–d). Because of only gradual change in angle proximo-distally (SI Fig. 1a–c), and the constant angles of the SBFs in numerous layers at a number of points along the axis (Figs 4, 5, 6), they are not considered to affect the anisotrophy of the cortex^18^.

SBFs fan out toward the barbs in waves (Fig. 2a–c, SI Fig. 5b), diverting to the proximal barbs first, succeeded by the next wave further inward to the next barb along the rachis, and so on (Fig. 2b). In a deep cross-section of the dorsal cortex of *Gallus gallus* (fungal delineated), the more-or-less constant SBF angles are noted in 20 + layers of SBFs (approximately a third of the dorsal cortical depth at mid-rachis length) (Fig. 5). There is no marked change in SBF orientation apart from a few that are only slightly disoriented, possibly during the sectioning process. Although the matrix or ‘glue’ has been stripped in this specimen, the regularity of the SBF angles is maintained by the close-knit association of the SBFs and the fact that these SBFs apparently belong to long continuous chains (see Discussion). This close-knit association of the SBFs contributes to the stiffness of the cortex. Their unique morphology, which includes nodes with hooks and rings, plays a major part in the design strategy of keeping the filaments locked together, somewhat similar to the functioning of the free syncitial barbules of the feather venation^18^,^19^,^33^. Significantly, a number of species of birds and flight types examined, from *Gallus gallus* to *Falco peregrinus,* all showed a similar SBF architecture of the cortex^18^. Careful examination of the longitudinal section of the cortex of *Falco tinnunculus* (Fig. 4a) shows approximately 6 layers of SBFs that appear to follow the same orientation. This is confirmed in vertical sections along the long axis of the rachis of *F. tinnunculus* and *F. peregrinus*. They are at least 10 SBF layers deep and all show constant SBF orientations (Fig. 6b,c respectively). Not only do these constant orientations coincide with the SBF organization in *G. gallus* (Figs 5 and 6a) but they can also be observed in the rachidial cortex of the retrices of the Toco toucan, *Ramphastos toco*^26^ (Fig. 6d).

The number of SBFs that enter the barbs from the rachis will depend on the location of the barbs on the rachis and size of the feather/bird. For instance in the black eagle, *Aquila verreauxii*, at the rachidial mid-length the number is estimated at ~10 SBF layers deep and ~15 wide (Fig. 2d). In a longitudinal section of the barb cortex of the much smaller *Falco peregrinus* the SBFs are ~9 SBF layers deep and ~6 wide at the midpoint length of the rachis^19^. Numerous SBFs are also noted entering the barbs at about mid-rachis-length in the sacred ibis, *Threskiornis aethiopicus* (SI Fig. 4d) The numbers would clearly vary with different barb cortical shape and size in different bird species^19^ but the relative values are likely to remain similar.

There was no detectable difference between the deviations of SBFs in the rachis closer to the leading edge compared with the trailing edge. If there are differences in the conditions mentioned above, they are subtle differences in the diverging SBFs and in the angles (Table 1).The ventral rachidial cortex also shows divergence of SBFs toward the barbs, as expected (SI Fig. 2e).

So as to avoid confusion it is necessary to say a few words on the fibre structure of the lateral walls of the rachis and barbs. It is important not to confuse the fibre structure (SBFs) of the rachidial and barb cortical tissue (dorsal and ventral walls) with the fibre structure of the epicortical tissue (lateral walls) of the rachis and barbs. In a number of bird species the microstructure of the epicortex of the rachis and barbs was previously investigated along most of their length and depth by microbial delineation (hydrolysis) as well as by conventional methods (including an entire angled-cross-section at the rachis-barb interface)^19^. Scanning electron microscopy revealed a novel system of crossed fibres (ranging in thickness from, 100–800 nm in diameter), oppositely oriented in alternate layers, over numerous layers^19^. Thus SBFs are not involved in the architecture of the lateral walls of the rachis and barbs at all. That novel finding of β-keratin fibre architecture in the lateral walls necessitated a modified diagram/model^19^ from that previously published^18^. It was also demonstrated that the epicortex continued uninterrupted from the lateral walls of the rachis to the lateral walls of the barbs^19^ (ref. 19, Fig. 2b).

### The cortical matrix

We get a rare glimpse up close of SBFs with the matrix or ‘glue’ intact in the rachidial cortex of the pygmy falcon, *Polihierax semitorquatus* (Fig. 7a). The feather was freshly moulted or dislodged (see Material and methods). As a consequence of the preparation technique of peeling a thin section from the rachis surface, fibrils in the SBFs may be disturbed, hence not leaving the smooth surfaces seen in microbial delineation. However, the advantage of longitudinal peeled sections of a layer of SBFs spanning the entire rachidial width is that we see for the first time with matrix intact the closely packed nature of the SBFs in the rachidial cortex–whether at 90° or deviating at ~80° to the barbs (Fig. 2a–c, SI Figs 1a–c, 2g and SI Fig. 5). Interestingly, the matrix forms just a thin coating of a glue-like substance that has a possibly granular consistency (Fig. 7a,b). In some areas, SBFs with ‘glue’ intact (Fig. 7b) are in the process of being degraded by fungi, a parasite we know to attack feathers in both living birds^34^,^35^ and dead^18^,^19^,^21^. Close by (a matter of microns), a SBF has been completely exposed after hydrolysis of the matrix by fungi (Fig. 7c). The SBF shows the typical hooked structure of the node seen in numerous bird species (cf. *Gallus gallus*, Fig. 5, inset), further evidence of the conservative nature of the microstructure of the feather cortex in birds across numerous species investigated. It is clear that this extraordinary cortical microstructure of the feather has evolved and been perfected over the millions of years of bird evolution.

## Discussion

Cracks can have catastrophic consequences in natural and man-made materials. The rachidial cortex of the feather is considered to be a brittle surface^26^, as opposed to the epicortex, which is ductile^19^. Griffith^30^ proposed that the much lower experimentally determined strengths of brittle solids such as ceramics, where there is little plasticity because of the difficulty of moving dislocations, were the result of the presence within the materials of a population of crack-like defects each of which was capable of concentrating the stress at its crack tip. Failure would occur when the stress local to the largest crack exceeded the theoretical fracture strength. As stated in the introduction, the potential for crack-like defects by abrupt fibre terminations is exceptionally high in the rachidial cortex unless there is an alternative way to deal with a reduction of SBFs in a cortex of diminishing volume proximo-distally. The results here have demonstrated a unique microstructural architecture of the feather rachidial cortex that averts such a potentially fatal flaw in the feather rachis by the diverting of hundreds of SBFs from the rachis to the barbs. A schematic drawing simplifies how this occurs (Fig. 8).

Previously, SBFs were reported in the barb cortex^19^ identical to those in the rachidial cortex^18^, and were assumed to ‘flow’ (at least some) from areas of the rachis directly adjacent to the barbs. In the present study I demonstrate for the first time in this context that the SBF architecture is considerably more complex. The SBFs start to divert or fan-out (Fig. 2a–c and SI Figs 2g, 5b) toward the barbs from close to the central region of the rachidial cortex at steep angles to the edges (Table 1). These fibres, which comprise a major fraction of the cortex (across a number of bird species), neither reach the more distal parts of the rachis nor are there any signs of terminations from the present investigations. Rather, they enter the barbs on either side of the rachis in large numbers (Fig. 2a–d, SI Fig. 5b). The present findings thus provide a key solution against a real possibility of fibres simply being terminated within the cortex and against the dangerous creation of populations of crack-like defects (Figs 1a, 8). In the ventral cortex too, although confirmed in just a few specimens here (also see Bode *et al*.^26^), it seems reasonable to assume a similar structure. As noted above, just a layer or two of SBFs at the rachidial edges/surfaces orient with the rachidial longitudinal axis (90°)(Figs 3a,b, 4a and SI Fig. 1a–c, arrows). This may serve a primary function of aerodynamic streamlining^19^.

Based on numerous selected sections of the rachidial cortex in many bird species we are able to reasonably project the high probability of continuous SBFs in the rachis which would avert the potentially fatal condition of hundreds of inherent cracks in the rachis. Particularly informative among these is a long segment of the rachis prepared by microbe delineation (SI Fig. 5a). The only evidence of breaks in the SBFs is where they are cut across by the dissecting blade in several places. Also informative in the segment is the presence of severely disrupted individual SBFs over a millimetre long dragged across the rachis intact (a testament to their toughness). This clear toughness indicates that SBFs are ideally predisposed to function as continuous tensile structures. Another particularly long section of 7–8 mm of the rachis shows a continuous system of fibres fanning out to the rachidial margins (SI Fig. 5b). Underscoring the prediction of continuous SBFs is a resolution of the hypothesis of the present study, “a means to eliminate this potentially catastrophic condition” of cracks in the feather rachis. The solution is a biomechanically ‘ingenious’ and novel architecture of the fibre organization of the rachidial and barb cortices whereby hundreds of SBFs are diverted to the barbs rather than being terminated. At a stroke hundreds of inherent cracks-like defects in the feather rachidial cortices are avoided with clear ramifications for bird flight^23^. However, unlike function-specific components in man-made structures, those in nature are usually multifunctional^36^,^37^. The need to divert large numbers of SBFs to the barbs rather than subject them to terminations may have other functional consequences for the feather microstructure. One such functional ramification will be discussed briefly below.

Feathers and the trunks and branches of plants are subjected to similar recurring mechanical stresses such as wind loading or other mechanical perturbations^31^,^38^,^39^. The most striking analogy of the SBF architecture in this respect is with the xylem tissue/vessels and fibres of plants. Xylem constitutes the major part of a mature plant stem or root. It is part of the vascular system that conveys water and dissolved minerals from the roots to the rest of the plant and, importantly, xylem also furnishes mechanical support. Xylem consists of specialized water-conducting tissues made up mostly of narrow, elongated, hollow cells. These cells may be of several types, including tracheids (the basic cell type), vessel members, fibres, and parenchyma.

Neely^40^ (1991), by injecting a water soluble dye, methyl violet, into the stems directly beneath branches of trees in a number of deciduous species was able to show a continuity of water transport from the xylem vessels in the stem to the distal xylem vessels in the branch. He was also able to show that dye injected beneath, but not directly beneath, a branch moved into the sides or top of the branch or into the stem. Recently, an anatomical model for junctions in trees has been outlined by Slater *et al*.^41^ based on visual observations of the grain patterns found at junctions of tree and shrub species and supported by CT scanning of bifurcations in common hazel (*Corylus avellana* L.). Significantly, this anatomical model emphasizes the importance of the xylem lying under the branch bark ridge as the main contributor to the bending strength of bifurcations^41^ and dense xylem formed at the apex of bifurcations plays a key function in preventing failure at the junction by supplying a higher bending strength^42^. While this analogy accepts there are differences with respect to remodelling etc. in trees compared to feathers, the fundamental point lies in the continuous lines of reinforcing xylem vascular systems that join the branches to the trunk in just the same sort of patterns as seen in the SBF system joining the barbs to the rachis in the present study, emphasized by the fact that the two analogues have very similar types of forces to withstand. As shown above the xylem vessel can be specifically tested because they also function in nutrient and water transport. It is clear, however, that regardless of the need for continuous vessels for nutrient support in trees, the need for reinforcement by a continuous system of vessels/fibres is fundamentally important. Xylem vessels provide by analogy strong support for a continuous system of SBFs in the feather cortical tissue of the rachis and barbs, as already implied by present microstructural findings (Fig. 2a–c, SI Figs 2g and 5), as a means to distribute the load from the slender barbs to the rachis.

A few points are worth mentioning with respect to the angles that the SBFs make when deviating to the barbs (Table 1). It is noteworthy that Cameron *et al*.^43^ found differences in orientation of keratin at the molecular level ranging from a higher angle “of supramolecular mis-orientation” to a lower angle (i.e., as they calculate, an angle of mis-orientation of 0 indicates perfect axial alignment). Hence, from the base of the rachis to the mid-length (50%) the fibrillar angle decreased from between 9.22–10.31° to 7.24–7.58°. These figures are roughly similar to the SBF angles reported here (Table 1; SI Fig. 1a–c) although at the time they^43^ could not have been aware that their molecular “mis-orientations” were in all probability associated with the SBFs only discovered later^18^. However, their increased fibre angles at 75% along the rachis is moot. Nevertheless, the significance of Cameron *et al*.’s^43^ x-ray diffraction studies can be seen in a new light in which their findings at the molecular level have validation and new impact at the microstructural level i.e. in the SBF angles reported here.

The biomechanical implications of most of the SBFs aligned longitudinally, (including those that divert at high fibre angles as shown here), is greater stiffness along this axis. We look at stiffness of the rachidial cortex in a little more detail.

A brittle structure is considered important in the feather rachidial cortex, which needs to be remarkably stiff to cope with the aerodynamic forces of flight^23^. Stiffness of a material, such as the feather cortex, is defined by its modulus of elasticity or Young’s modulus (*E*), which is the ratio of the applied stress to a body over the strain. Thus the Young’s modulus is a measure of the ability of a material to withstand changes in length when under lengthwise tension or compression. High stiffness was confirmed in a biomechanical analysis in the bulk cortex of the rachis of the Toco Toucan, *Ramphastos toco*^26^, where the SBFs are oriented at constant, high angles longitudinally (Fig. 6d). The authors observed brittle failure which was consistent with patterns of near-periodic aggregations similar to those observed previously^18^,^19^ and in the present study. On the other hand in some plants, cellulose fibres aligned in layers with different orientations to the long axis were shown to contribute to a remarkably large range of moduli and stiffness in various layers in the plant cell wall. While ductile structures as in some plants^38^ may be important to enable bending so as to respond to wind forces, Bode *et al*.’s^26^ mechanical tests, supported by morphological analyses (Fig. 6d), point against varying orientations of SBFs in feather cortical microstructure. Butler and Johnson^32^ also considered that longitudinal fibres in the cortex coincides with increased flexural stiffness during flight; but by allowing for twisting when loaded with dangerously high forces. Subsequently, it was found that the feather has a special way of dealing with torsional forces i.e., through the lateral walls, which are ductile surfaces^19^.

Interestingly, higher stiffness associated with layers of structural fibres predominantly in a single orientation was also noted in the dorsal and caudal fins of one of the fastest swimming marine vertebrates in the oceans, the white shark, *Carcharodon carcharias*. This structural architecture of high fibre angles oriented at the same angle in numerous layers was considered to have functional implications connected with control surfaces dedicated to stiffness and stability during high-speed locomotion in a fluid medium^44^,^45^,^46^.

Near periodic longitudinal orientations of the SBFs at approximately 80 to 90° (Figs 2, 3, 4, 5, 6 and SI Fig. 1a–c) in multiple layers enable a unique bonding mechanism with adjacent SBFs in both 2- and 3-dimensional planes. This is essentially achieved by the nodes and hooks which function ideally when the SBFs are aligned in a close, tight formation (e.g. Figs 2 and 4, 5, 6, 7 and SI Figs 1, 3a, 5). This grappling/bonding mechanism would clearly also facilitate layer cohesion, which in contrast would be disrupted if SBFs were oriented in different directions in different layers. We have previously seen^22^ the potential for a larger load distribution in 2- and 3-dimensional planes of SBFs in the rachis, which is consistent with the “brick-bridge mortar” structural model^47^. As shown above the only exceptions are in a thin outer layer of tangentially oriented SBFs (around the hoop), which serves to prevent failure by the Cook-Gordon mechanism^19^,^23^,^31^ although they too are tightly associated and parallel^18^.

Previously it was proposed that the dogbone shape of the SBFs functions to prevent or minimise ‘pull out’ of the SBFs from the surrounding matrix and improve the transmission of forces– analogous to the structure and function of steel rebars used to reinforce and improve elasticity in concrete in high-rise building construction^18^,^22^. However, while the analogy holds good, in other respects because of the high ratio of SBFs to matrix compared to rebars to concrete, a further analogy in this context is appropriate. Engineers working on nanotubules^48^ demonstrated that a thin layer of glue improved the tensile stiffness of the material by improving lateral slippage. Although unknown to them at the time we see a similar form of bonding of SBFs in the feather cortex by the matrix (Fig. 7). But we also see that the architecture of SBFs in the feather is more sophisticated compared to the engineered tubules i.e., with respect to the dogbone shape (aided frequently by hooks), which functions in fibre pull-out (Figs 4, 5, 7 and SI Fig. 3). This must be a tribute to over 150 million years of feather evolution in response to extreme aerodynamic stresses involved in bird flight.

Striking images in a narrow zone of the cortex of the pygmy falcon show the SBFs and matrix (i) intact and tightly bonded, (ii) in the process of being fungal degraded and, (iii) fungal degraded (Fig. 7). A schematic cross section of the non-degraded SBFs and biodegraded ‘matrix was presented in our earlier study 18 in an attempt to hypothesize the structures biodegraded in the amorphous matrix. They are postulated as: (i) the residual cytosol of keratinocytes presumably housing effete organelles and perhaps cytoskeletal elements—all degraded along with corneous envelope; (ii) interdigitating plasma membrane of the original keratinocytes with associated corneous envelope proteins. This may prompt future research into the phenomenon of selective βkeratin biodegradation in the feather cortex between the matrix and the SBFs.

Different approaches have been investigated to model the behaviour of cellular materials as in the pioneering developments of Gibson and Ashby^49^ involving basic deformation mechanisms. In X-ray tomography applied to the characterization of cellular materials, Maire *et al*.^50^ proposed that the microstructure of the constitutive material is firstly important. This aspect of the structure they state is generally inspected by means of microscopy techniques such as optical or scanning electron microscopes with a suitable resolution. Most authors of more recent work endorse this view as pertinent to studies involving mechanical tests^26^,^31^,^51^,^52^,^53^. However, a recent study by Laurent *et al*.^27^, mentioned in the introduction, proposed that their findings show “multiple laminae of differentially oriented fibres” in the cortical tissue of the rachis and “resulting mechanical anisotropy.” See my contrary results above. Given a novel use of nano-indentation and X-ray tomography in this context the authors regrettably did not support their interpretations with microscopy. Indeed, as we have seen from the present study of strikingly different results, validation by microscopy (e.g. SEM), as recommended by numerous authors, given the X-ray tomography images are of insufficient resolution, is warranted.

The field of biomimetics is relatively new^36^,^54^,^55^,^56^ but has been receiving increasing attention in recent years. Biological materials are complex composites that are hierarchically structured and multifunctional. Their mechanical properties are often outstanding, considering the weak constituents from which they are assembled^37^. Structures in nature have evolved over millions of years (frequently hundreds of millions) and because of their multifunctionality are difficult to resolve or break down into simpler components that could help in engineered materials. Whereas current engineering designs benefit from simplicity, future ones might be more sophisticated with a much wider performance envelope and broader range of applications inspired by biology’s vastly different scales of architectural organization and robust multi-functionality^36^. As biologists and evolutionists we hope to help untangle some of the complexities of natural materials by extensive investigations and to collaborate with materials scientists and engineers with the ultimate goal of mimicking them in synthetic systems. The contribution of bird feather microstructure to biomimetics, as predicted^22^, recently received affirmation from engineers. Kazemahvazi *et al*.^57^ named their new 3-D assembled carbon nanotubule beam as the “bird feather rachis-like beam” i.e. as a “highly efficient naturally occurring fibrous structure” that “can diagrammatically be treated as a beam with webs and faces (3)[ref. to Lingham-Soliar^22^]”.

## Material and Methods

### Ethics Statement

All methods were carried out in accordance with relevant guidelines and regulations. No animals were harmed during the course of this study. Most feathers used were freshly moulted and provided by Umgeni Bird Park, Durban and African Dawn Bird and Wildlife Sanctuary, Port Elizabeth. Feathers of *Falco peregrinus* were obtained from a bird that had died from natural causes in the wild and those from *Gallus gallus* from “Rainbow Chickens, Pietermaritzburg” from an earlier study^19^.

### Data

I tested a number of birds species in the present study: the domestic chicken, *Gallus gallus,* black eagle, *Aquila verreauxii,* common kestrel, *Falco tinnunculus, sacred ibis, Threskiornis aethiopicus,* pygmy falcon, *Polihierax semitorquatus,* mute swan, *Cygnus olor*, peregrine falcon, *Falco peregrinus*, scarlet ibis, *Eudocimus ruber*, golden pheasant, *Chrysolophus pictus*, spur-winged goose, *Plectropterus gambensis*, the blue and yellow macaw, *Ara ararauna* and the marsh harrier *Circus ranivorus*. All feathers obtained were frozen immediately if not used at the time of acquisition. *Gallus gallus* has proved useful as a basis for certain types of investigations as many authors have found^18^,^28^. It was invaluable in my own work for understanding feather cortical microstructure because it is easiest to get replicate samples from fresh birds^18^,^19^. Also I believe there are unlikely to be any significant internal microstructural differences of the feather rachis and barbs during domestication^27^ compared with wild birds. While chickens do not engage in sustained flight, they do regularly fly up to their perches as well as engage in periodic burst flights, which are often more strenuous^58^,^59^ parts of the flight cycle. Rather, it is more likely that macrostructural changes would place a premium on their ability for sustained flight. Hence, no birds were treated as definitive for the structures investigated or in the order of investigation. However, because of greater size of feathers (given delicacy of the tests) it was more convenient to perform more sections along the rachis and at different depths in *Aquila verreauxii* and *Cygnus olor.* In support of the idea of decreasing SBFs along the rachidial cortex proximo-distally, two sections were made from fungal degraded rachises in *Gallus gallus* (at ½ - and ¾- rachis lengths and ~1/3 and 2/3 depths respectively).

### Techniques

Now that we understand the fibre hierarchical structure of the feather cortex, delineating the fine structure of the syncitial barbules as originally necessitated by microbes, is not always necessary but, depends on the questions being asked and details required. Other quicker techniques were demonstrated for investigating feather microstructure by SEM with hindsight wisdom^19^,^20^,^21^. Because keratin is the toughest natural polymer it is difficult to separate the fibrous structure from the binding matrix, hence none of the methods are simple, but this must be underscored by the fact that we are looking for real answers to a complex problem.

I used a number of preparatory techniques for SEM examination. The most involved technique is by fungal degradation and resin embedding^18^,^19^,^21^. Longitudinal sectioning of fresh feathers frequently leaves split, partial fibres and fibre debris but with patience, perhaps requiring 20 + sections, the method is capable producing sections of relatively complete SBFs with exquisite detail (certainly not as involved as fungal hydrolysis). Peeling thin sections of the rachis is a useful and simple technique for wide-angled views (e.g. of the complete rachidial width) of SBF organization, which workers may find perhaps the most accessible preparatory method for many investigations into feather structure (described below). SBFs should be easily discernable by their size alone even when bonded with other fibres and at lower magnifications. Freeze fracture is a further method. The method on fungal delineating will not be repeated here. Note fungal hydrolysis was performed in the lab on bird cadavers^18^,^19^,^21^.

#### Peeling

Longitudinal sections are peeled off about 1 cm lengths of the rachis. It is done under a binocular microscope. First, a nick is made in the cortex using a scalpel or microtome blade. Next, a pair of fine forceps is used to strip off a thin layer of the cortex along the length of the section. Peeling works quicker in some respects than manual sectioning or microtoming because the separation occurs at the points of least resistance i.e. along the fibre surfaces, usually exposing whole SBFs in a wide (frequently the entire rachidial width) layer with sometimes little disturbance or ‘pull-out’ of SBFs. The other advantage over microtoming or manual splitting with a blade is that SBFS are frequently cut through and unrecognizable in the latter methods. This method is less daunting than microbial delineation and probably the most accessible method for most workers with results speaking for themselves (e.g. Figs 2, 3, 7 and SI Figs 1, 5b).

#### Sectioning

Manual cutting using a blade is equally simple. A one centimetre section of the rachis is split manually (longitudinally) using a scalpel blade, but as mentioned above there is no guarantee that there will immediately be an informative section showing recognizable SBFs, which may need numerous sections. However, when appropriate sections are obtained, the results may be among the best thus far achieved.

Freeze-fracturing is a commonly used technique that may give good size delineation of the SBFs. A section of the rachis is submerged in liquid nitrogen and then manually fractured. However, the distinctive swollen nodes, with hooks and/or rings are frequently not easily recognizable. Also, when trying to determine SBF angles it is quite difficult from a standard cross-section because of the near circular SBF. Hence, I have preferred an angled cross-section (showing partial longitudinal and cross-sectional views) and/or longitudinal sections.

SBF angles were measured using the program Uthesca (Table 1). Ninety degrees is considered here as the longitudinal axis of the feather rachis.

### Technological preferences

The choice of SEM relates to the focus of the present study namely the hierarchical fibres i.e. syncitial barbules and their role as building blocks of the rachidial cortex microstructure. Hence, the focus of the present study does not concern itself with their internal composition at ultrastructural or molecular levels, which may benefit from TEM. The latter can show many characteristics of the sample, such as molecular and atomical structure as well as crystallographic information. SEM images on the other hand are 3-D and accurate representations at a lower level (micron) of investigation and give accurate images for direct microstructural analyses. TEM pictures are 2-D and require interpretive reconstructions for more 3-D representations of larger visual fields. Furthermore, reconstructions from TEMs of unknown microstructure can be problematical when making 3-D interpretive reconstructions. For this study for testing the present hypothesis, x-ray diffraction was considered unnecessary and less helpful than direct, visually acute 3-D imaging by SEM. Furthermore, I do not believe we can expand on Cameron *et al*.’s^43^ findings in this respect. Their findings (see Discussion) at the molecular level in this context can be seen in a new significant light when compared with my findings at the microstructural level.

## Additional Information

**How to cite this article:** Lingham-Soliar, T. Microstructural tissue-engineering in the rachis and barbs of bird feathers. *Sci. Rep.* **7**, 45162; doi: 10.1038/srep45162 (2017).

**Publisher's note:** Springer Nature remains neutral with regard to jurisdictional claims in published maps and institutional affiliations.

## Supplementary Material

Supplementary Information

## Figures and Tables

**Table 1:** Syncitial barbule fibre angles in the rachidial cortex on either side of the midline as they diverge to the barbs.

species	Number of	Fibre angle	Std. Dev.
	Measurements *n*	Mean	
*Falco tinnunculus*	8	79.7	2.36
*Cygnus olor*	12	76.7	3.8
*Falco peregrinus*	11	79.9	1.7
*Gallus gallus*			
*i) Fresh, hand cut*	15	82.1	1.95
*ii) biodegraded*	10	80.2	4.8
*Circus ranivorus*	8	78.2	4.75
*Polihierax semitorquatus*	10	81.7	4.82
*Aquila verreauxii*			
*i*) 40% rachis L	8	75.66	6.35
ii)3/4 length	8	79.7	2.793
iii) 7/8 length	12	82	2.92
*Threskiornis aethiopicus*	12	77.3	3.91
*Aquila verreauxii,* mid-length 50%	9	78.9	4.7
*Eudocimus ruber*	11	78.41	2.52

Ninety degrees equals the longitudinal axis.
