# Supplementary material for: Microstructural tissue-engineering in the rachis and barbs of bird feathers
Source: Sci Rep. 2017 Mar 27;7:45162. doi: 10.1038/srep45162 (PMC5366878; doi:10.1038/srep45162)
Supplement: Supplementary Information [file srep45162-s1.pdf]

Supplementary Information .

Microstructural tissue-engineering in the rachis and barbs of bird feathers.

Author: Theagarten Lingham-Soliar

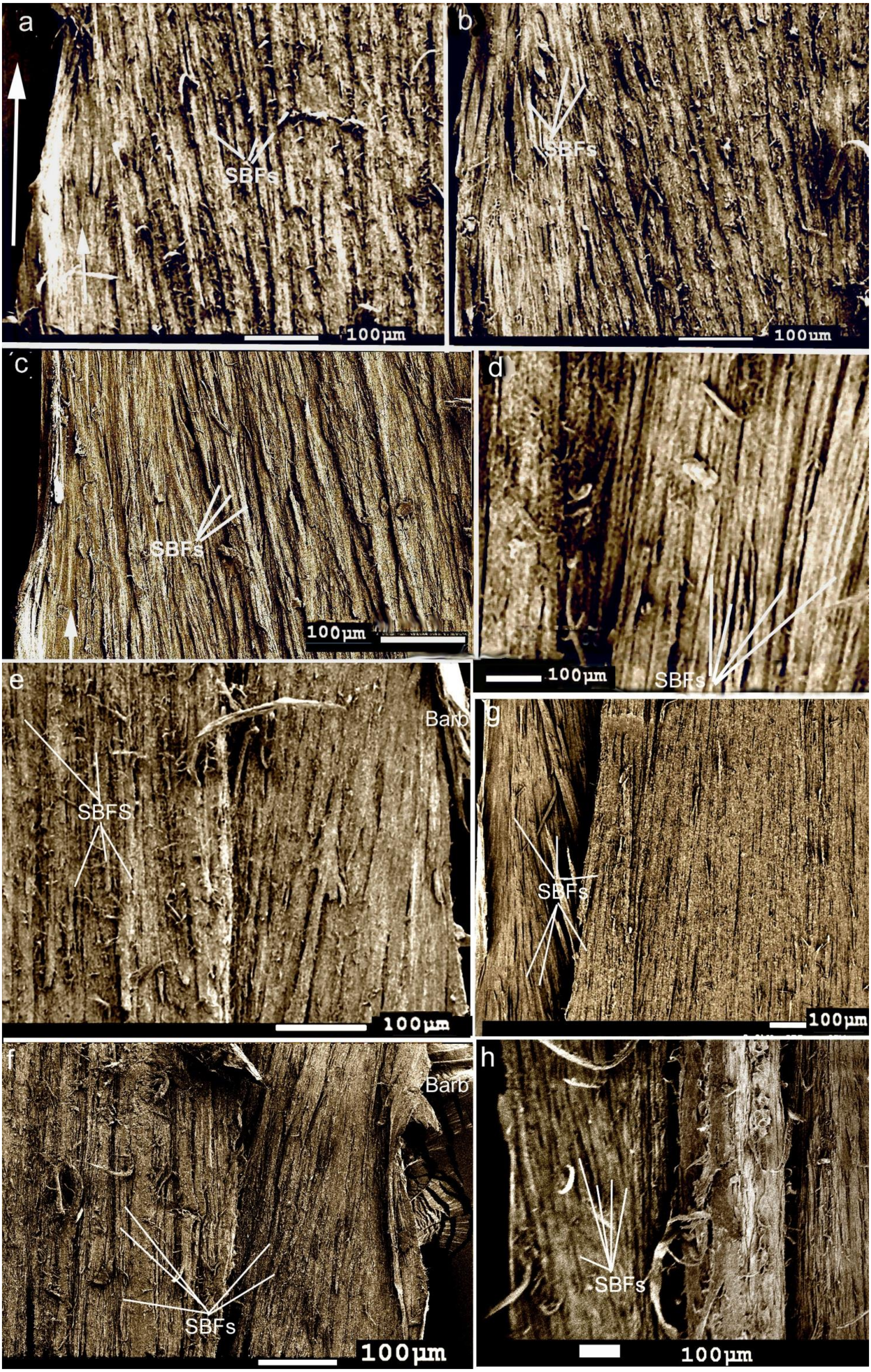

a
SBFs
100µm
b
SBFs
100um
c
SBFs
100µm
d
100µm
SBFs
e
Barb
SBFS
100µm
g
SBFs
100µm
f
Barb
SBFs
100µm
h
SBFs
100µm

SI Fig. 1. SEM of primary flight feather of the black eagle, *Aquila verreauxii* (longitudinally peeled sections of rachis to expose cortical SBFs). (a-c, left side SBFs in line with top surface of the barbs) at 7/8 i.e. near tip (1mm wide) to ¾ (1.5 mm wide) to 2/5 (2.5 mm wide) rachis length. Note fibre angles become steeper distally (see table 1). (d) Mid-rachis, right side, SBF in line with top surface of barbs. (e) Right side, at 7/8 length, SBFs near cortex surface. (f) Right side, at 7/8 length, SBFs at bottom of cortex just before the pith. (g) Left side, near base of rachis at 1/5 length, SBFs near the surface of cortex. (h) Left side, base of rachis at 1/5 length, SBFs near the bottom of the cortex, indicated by cortical ridge base,

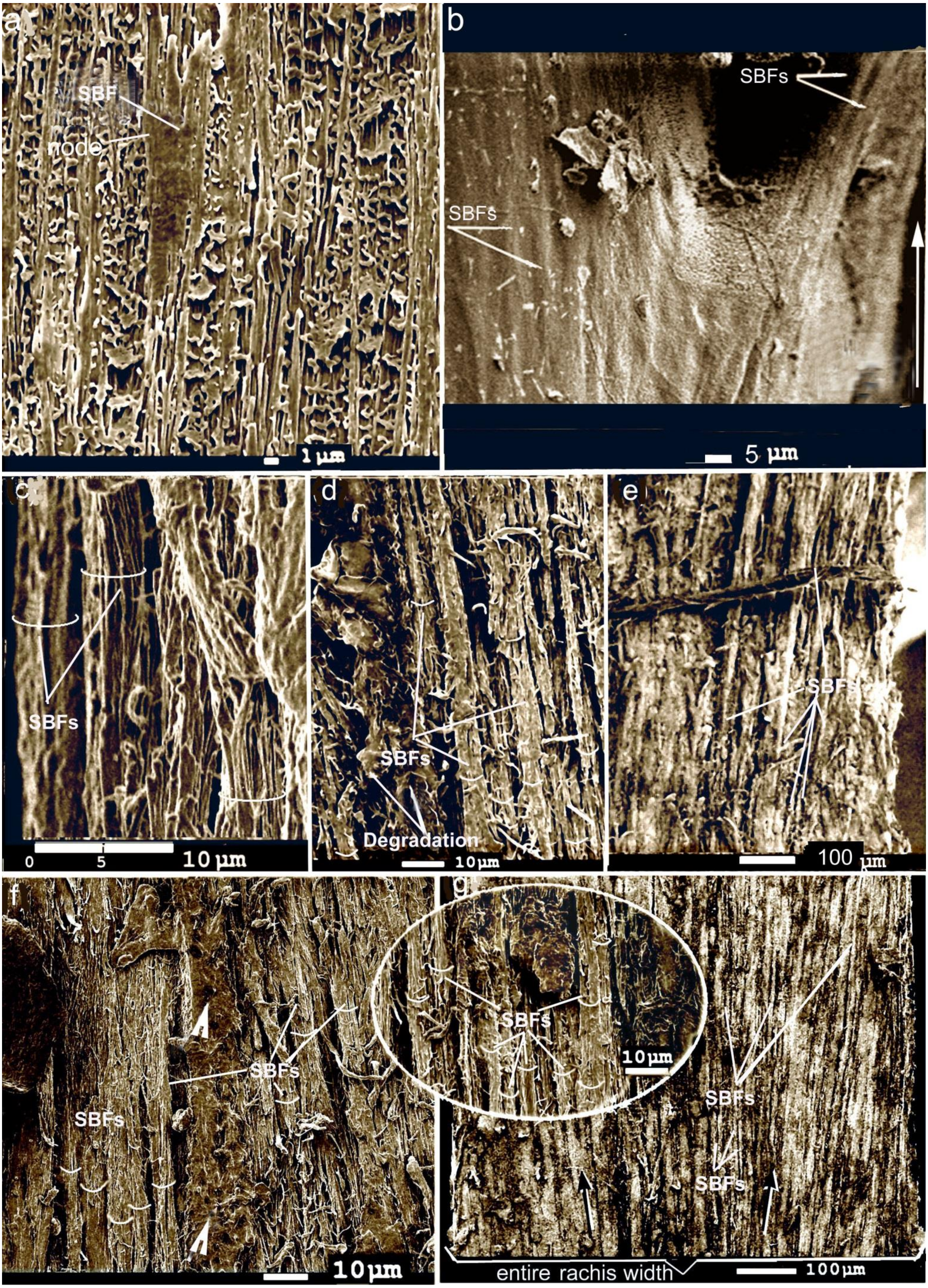

SI Fig. 2. SEM. LS. Primary flight feathers. Rachis cortex. SBFs. (a, c, d, f). Mute swan, *Cygnus olor*. (a) Mid-length, right, near surface. Microtomed section of SBFs. Most fibres have been cut through. (b) *Gallus gallus*. Mid-length, right surface (slight microbial delineation) show SFBs starting to diverge to barbs. Right arrow shows longitudinal axis

for all sub-images. (c) Tip, left edge, level with barbs showing SBFs. (d) SBFs from mid-length just above medullary pith. Note, degradation label, points to fungal hydrolysing acids. (e) Black eagle. Mid-length, ventral section, right. (f) SBFs of *Cygnus olor,* mid-length just above medullary pith. Hemicircles demarcate some SBFs. Arrowheads show fungal hydrolysis noted in (d). (g) Pygmy falcon *Polihierax semitorquatus*, mid-length, entire width, near surface (see detailed section in text Fig. 3c). The close association of the fibres and the left and right orientation of the fibres starting from near the mid-area is noteworthy. Inset, shows detail of some of the syncitial fibres on left side of rachis.

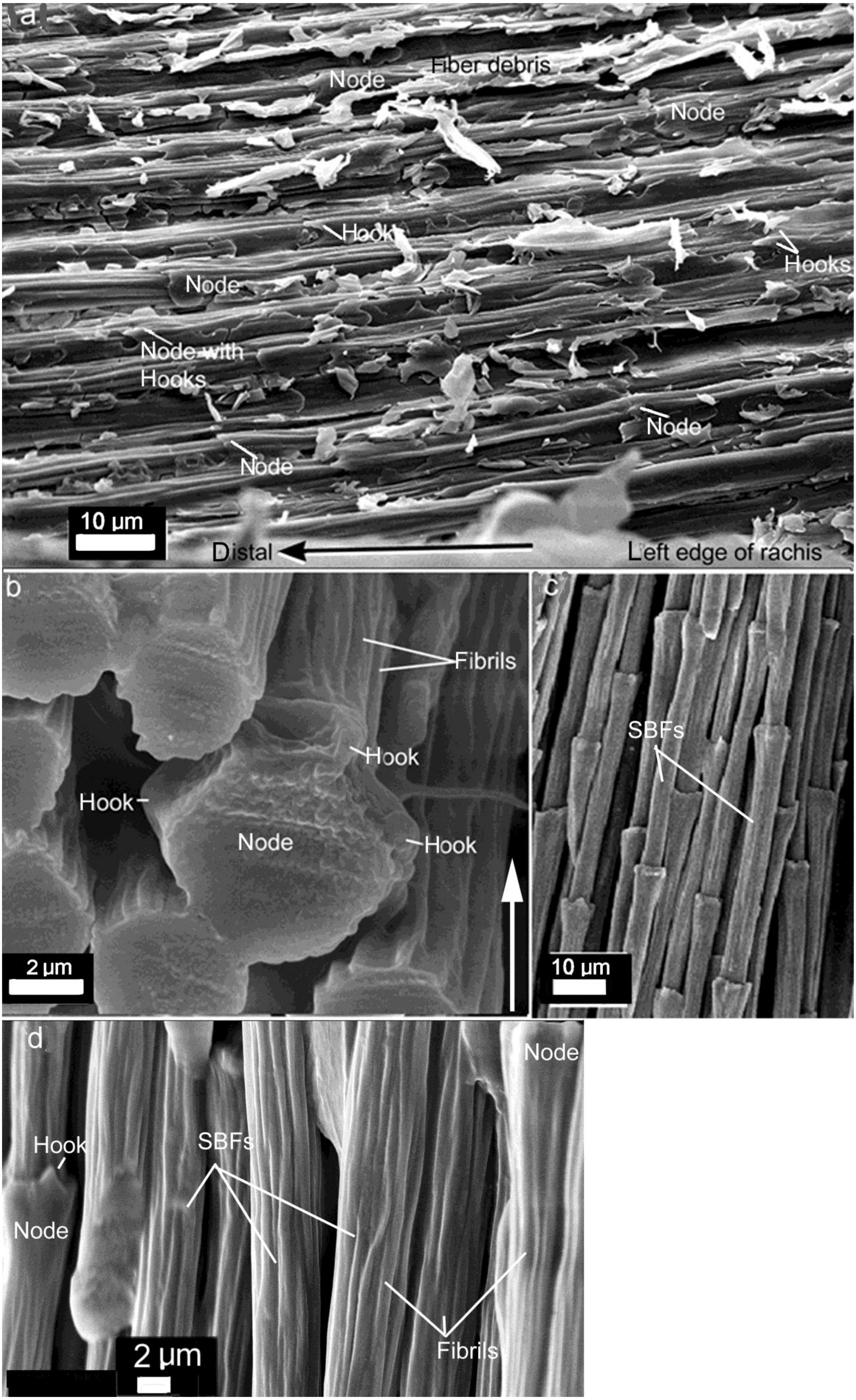

a
Fiber debris
Node
Node
Hook
Hooks
Node
Node with
Hooks
Node
Node
10 µm
Distal
Left edge of rachis
b
Fibrils
Hook
Hook
Node
Hook
2 µm
c
SBFs
10 µm
d
Node
Hook
SBFs
Node
Fibrils
2 µm

SI Fig. 3. SEM of primary flight feather of *Gallus gallus.* Rachis cortex. SBFs. (a) Native feather. Mid-length, left. Hand-sectioned using scalpel blade (LS). Note the close packing of the fibres, although some have been destroyed during sectioning. Arrow shows longitudinal axis and direction of the rachis from base. (b-d) Microbial delineated. (b) Magnified 18,500x. Partial cross- to longitudinal-section, showing different layers of the syncitial barbule fibres in 3-dimensions. The central SBF is cut across the node, the thickest part of the SBF. (c) Mid-length, right. (d) Magnified 8000x. Note, large fibrils and nodular hooks. Arrow in (b) represents approximate longitudinal axis of the rachis in (b)-(d).

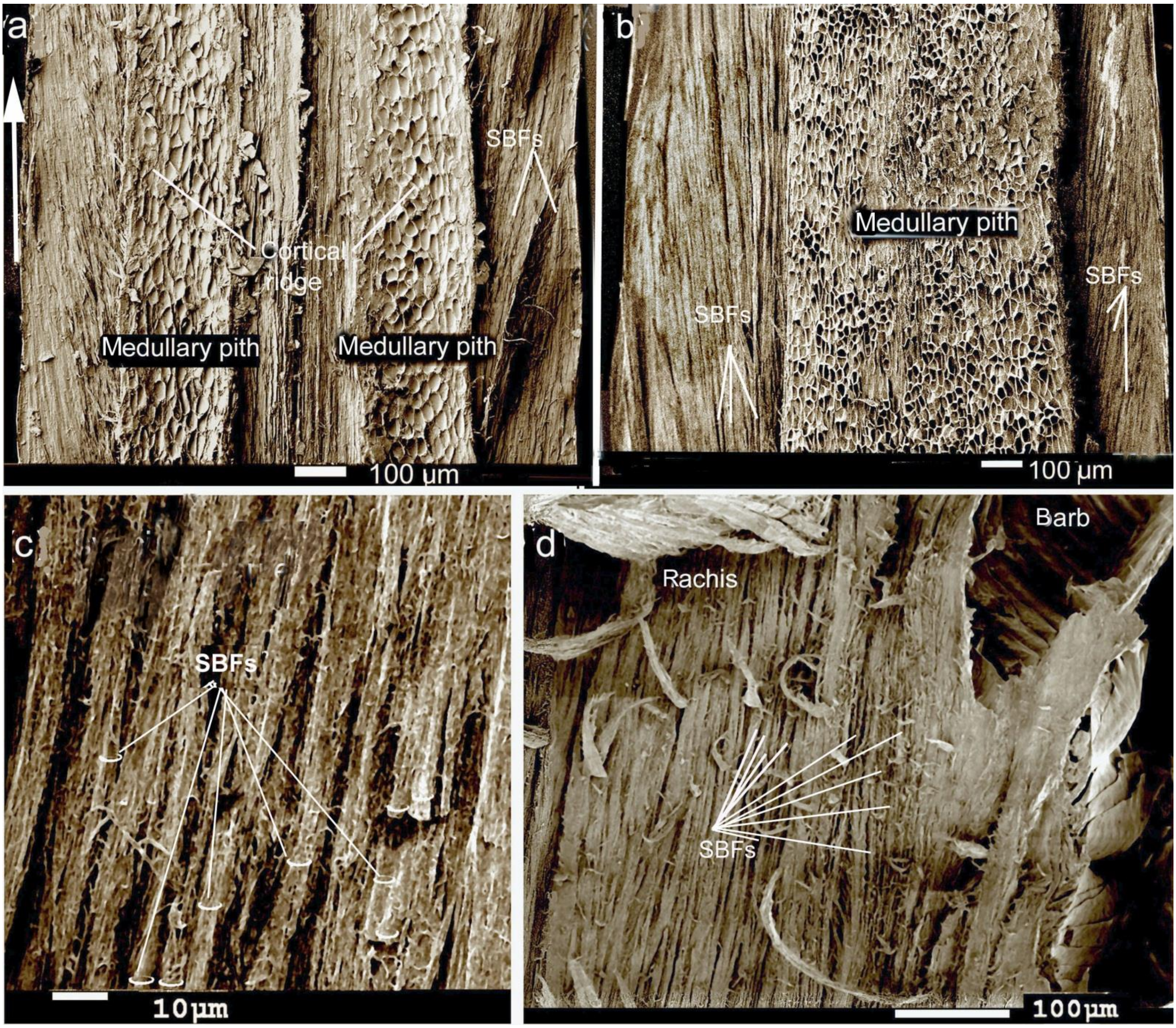

SI Fig. 4. SEM of primary flight feathers. Rachidial cortex. SBFs. (a) Golden pheasant, *Chrysolophus pictus* showing entire width of rachis at mid-length. Section is peeled fairly deep and reveals the beginning of the medullary pith on either side of the cortical ridge. Arrow, shows longitudinal axis of rachis in all figures.(b) Scarlet ibis, *Eudocimus ruber.* Section from deep in the rachis at mid-length, showing entire width. Here the medullary pith is not cleaved by a cortical ridge. (c) Showing detail of SBFs from (b). Hemicircles demarcate SBFs. (d). Sacred ibis, *Threskiornis aethiopicus*. Mid-length of the rachis peeled to show a wide-angled view of SBFs entering a barb. Part of the barb cortex was sheared during the process.

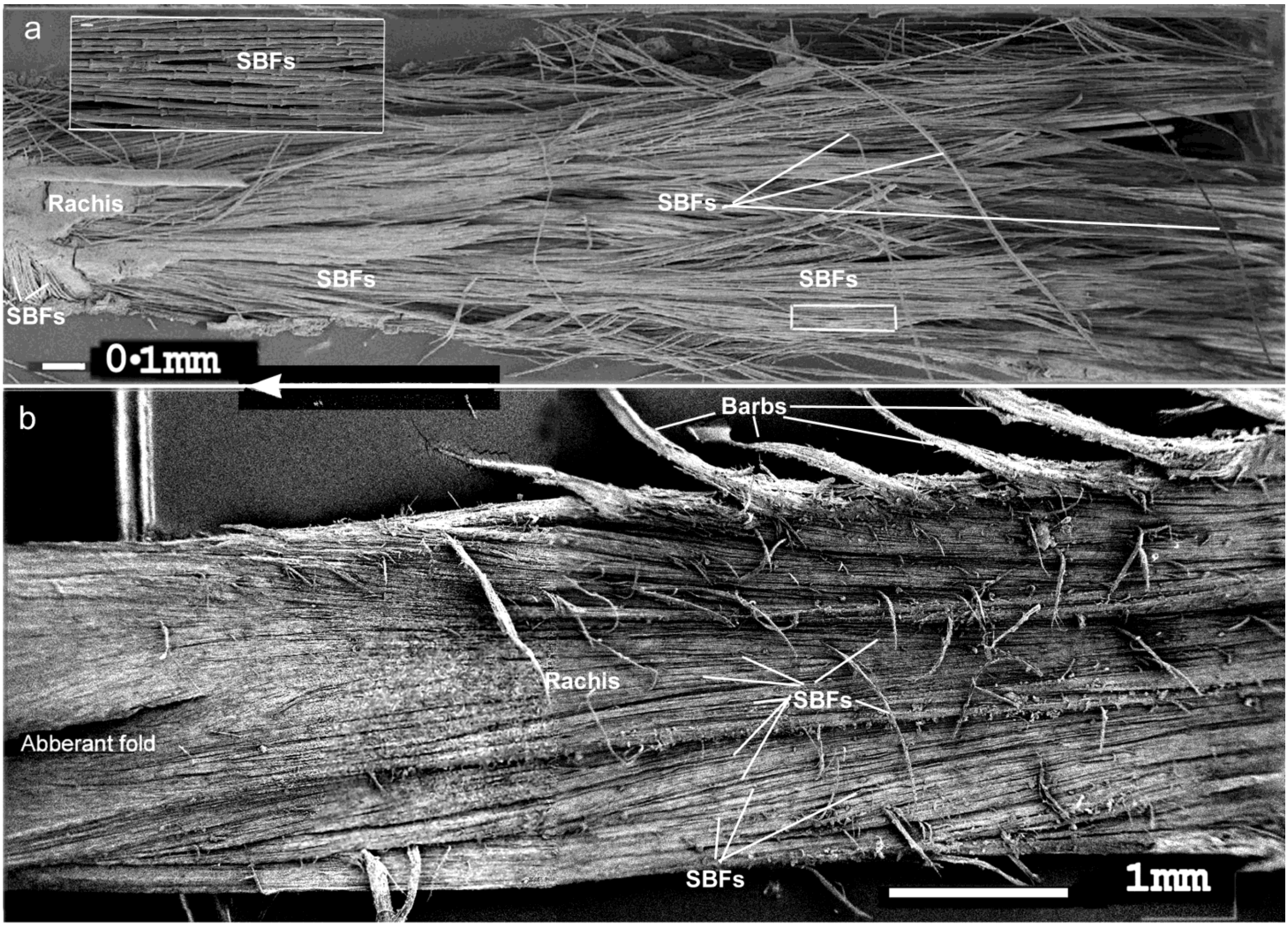

SI Fig. 5. Mid-length Rachis. Cortex showing long tracts of SBFs. (a) *Gallus gallus* 4-5 mm longitudinal section of microbially degraded flight feather rachis. Slicing and disruption of SBFs is evident during sectioning but also numerous long SBFs intact despite forceful displacement. White rectangular outline containing SBFs shown enlarged in inset. (b) *Aquila verreauxii.* Prepared by peeling. Mid-length, plane of barbs.Long filaments of SBFs are noteworthy as they diverge to the barbs in a long segment of the rachis in a novel view at the extreme limits of SEM that includes visualization of SBFs at maximum operating SEM section length. Despite the relative forcefulness of the peeling process as testified by a number of pulled-out SBFs, the majority are remarkably intact and in place. Arrow adjoining both images shows longitudinal axis of the rachides and direction from base.
